# Silicon Supplementation for Bone Health: An Umbrella Review Attempting to Translate from Animals to Humans

**DOI:** 10.3390/nu16030339

**Published:** 2024-01-24

**Authors:** Abby Pritchard, Brian D. Nielsen

**Affiliations:** 1 Department of Animal Science, Michigan State University, East Lansing, MI 48824, USA; bdn@msu.edu; 2 Regulatory and Nutritional Compliance, Mars Petcare, Franklin, TN 37067, USA

**Keywords:** silicon, bone health, mineral metabolism, silicon supplementation, bone mineral density, orthosilicic acid, aluminosilicate

## Abstract

Studies have attempted to demonstrate the benefits of silicon on bone health using a wide range of Si amounts—provided in the diet or through supplementation—and several different animal species. Previous studies in humans have also demonstrated a positive correlation between Si intake and bone health measures. The aim of the current review is to determine the effective levels of Si intake or supplementation that influence bone health to better inform future study designs and guidelines. Articles were identified using one of two search terms: “silicon AND bone” or “sodium zeolite A AND bone”. Articles were included if the article was a controlled research study on the effect of Si on bone health and/or mineral metabolism and was in English. Articles were excluded if the article included human subjects, was in vitro, or studied silica grafts for bone injuries. Silicon type, group name, Si intake from diet, Si supplementation amount, animal, and age at the start were extracted when available. Dietary Si intake, Si supplementation amount, and the amount of Si standardized on a kg BW basis were calculated and presented as overall mean ± standard deviations, medians, minimums, and maximums. Studies that left out animal weights, amount of food or water consumed, or nutrient profiles of the basal diet were excluded from these calculations. Standardized Si intakes ranged from 0.003 to 863 mg/kg BW, at times vastly exceeding current human Si intake recommendations (25 mg/d). The lack of data provided by the literature made definitively determining an effective threshold of supplementation for skeletal health difficult. However, it appears that Si consistently positively influences bone and mineral metabolism by around 139 mg Si/kg BW/d, which is likely unfeasible to attain in humans and large animal species. Future studies should examine this proposed threshold more directly and standardize supplemental or dietary Si intakes to kg BW for better study replication and translation.

## 1. Introduction

### 1.1. Role of Silicon in Bone Development

Silicon plays a role in bone and cartilage development. Early studies demonstrated that basal diets deficient in Si reduced the overall growth in chicks and rats [1,2] and altered long bone and skull formation in chicks [3,4], producing more-porous, less-mineralized bone. Typically, Si associates more with the organic matrix of bone and soft tissue [5,6], and declining Si concentrations in connective tissue may also be an indicator of decreasing collagen content [7]. In vitro, Si stimulates the production of type I collagen and mineralization activity in osteoblasts [8,9]. Through its association with type I collagen, Si contributes to the early calcification of bone’s organic matrix by providing a low solubility matrix to attract and contain other ions, such as Ca, at the organic–inorganic interface [10].

### 1.2. Effects of Silicon Supplementation

While deficiency proves harmful, supplementation may be beneficial. Silicon supplementation in the form of sodium zeolite A has increased distances accumulated during training before bony or soft-tissue injury in horses [11], possibly due to reduced resorption during periods of disuse or other alterations in bone turnover [12]. Silicon supplementation in calves has increased hydroxyproline content, an early marker for collagen formation, in skin [13], since Si stimulates type I collagen synthesis [8,14]. During both normal conditions and Ca deficiency, Si supplementation inhibits bone resorption [15] by reducing osteoclast formation and activity [16] as well as increasing osteoblastogenesis [17] and osteoblast activity [8,9,18]. The direct effects on bone cells and collagen synthesis could assist in cartilage repair and bone strength and demonstrate the importance of Si within the diet. In humans, silica-based nanomaterials have been used for bone tissue engineering and repair [19] due to these effects, and higher Si intake has been associated with greater bone mineral density [20,21,22]. However, the effects of Si supplementation above adequate intakes on bone and cartilage measures have been lacking. 

Silicon can also alter mineral metabolism. These effects can be beneficial when it comes to binding metals like aluminum to generate aluminosilicates to prevent absorption and harmful accumulation in tissues [23]. The effects of Si on serum Ca concentrations in supplemented animals are mixed, with some studies showing greater concentrations [13,24], but more recent studies show no difference or decreased Ca concentrations with Si supplementation [25,26]. Magnesium retention [27] and serum concentrations [24,26] can also be reduced with Si supplementation. However, these alterations in serum or plasma minerals do not always translate into changes in mineral concentrations in bone or soft tissue. Silicon in the diet increases Ca concentration in bone above amounts in a deficient diet [28,29], but other studies supplementing Si on top of an adequate diet show no changes in the Ca concentration of bones [26,30]. By altering both mineral metabolism and collagen synthesis, Si supplementation may increase bone density [5,15] and strength [30,31,32]. All these direct and indirect effects play a central role in improving and maintaining bone and cartilage quality during growth and later life, demonstrating Si’s importance as a micromineral.

### 1.3. Clinical Relevance

**Figure 1:**
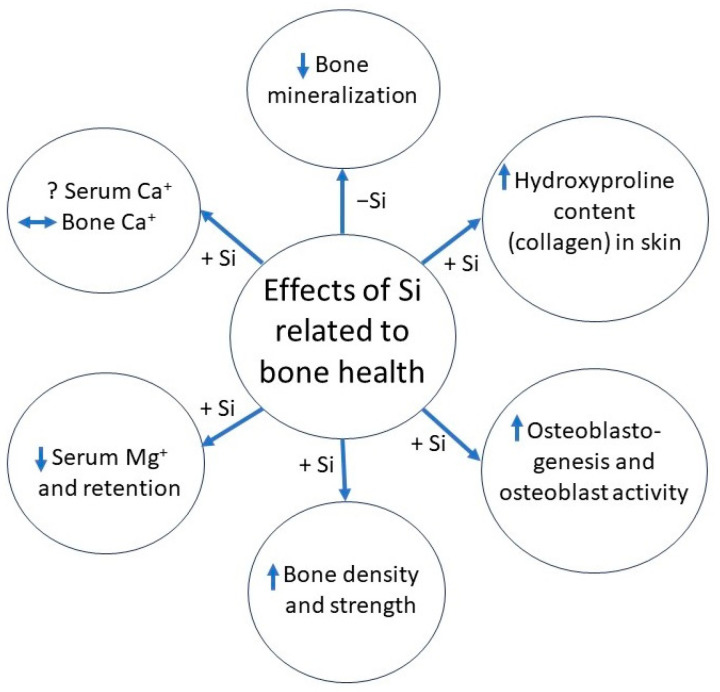
Summary of effects of silicon supplementation (+Si) or deficiency (−Si) on outcomes related to bone health. Direction of arrow indicates either increase, decrease, or no change in measured outcome.

A visualized summary of Si effects related to bone health from previous animal experiments has been provided in Figure 1. In humans, Si intake ranges between 12 to 62 mg/day, depending on diet and location [33]. Greater dietary Si intake correlates with greater bone mineral density in men and pre-menopausal women [34], indicating that Si plays an essential role in bone health in humans as well as animals. However, dietary Si retention from various sources appears relatively low, with less than 10% accounted for in serum and around 40% excreted in urine, though a full study of Si balance was not conducted [35]. In combination with Vitamin D and calcium, supplemental Si can positively influence bone turnover and increase femoral BMD in post-menopausal women [33], indicating the need to establish an effective threshold for supplementation to improve clinical outcomes. 

Despite the promise of Si to address various musculoskeletal issues in animals and its correlation with better bone health in humans, experimental results can be mixed. A wide range of animals have been used in studies to demonstrate the essentiality or benefits of Si with an even wider range of Si amounts provided in the diet or through supplementation. The aim of the current review is to determine the effective levels of Si that influence skeletal health to better inform future study design and guidelines. 

## 2. Methods

### 2.1. Inclusion/Exclusion Criteria

Articles that met the following inclusion criteria were included: (a) The article was a controlled research study on the effect of Si on bone health and/or mineral metabolism and (b) the article was in English. Articles were excluded if they met the following criteria: (a) The article included human subjects, (b) the article was in vitro, or (c) the article studied silica grafts for bone injuries.

### 2.2. Search Strategy

The authors identified articles for this review from PubMed, published from January 1967 to April 2023, using one of two search terms: “silicon AND bone” or “sodium zeolite A AND bone”, due to the authors’ research experiences. 

### 2.3. Article Selection

**Figure 2:**
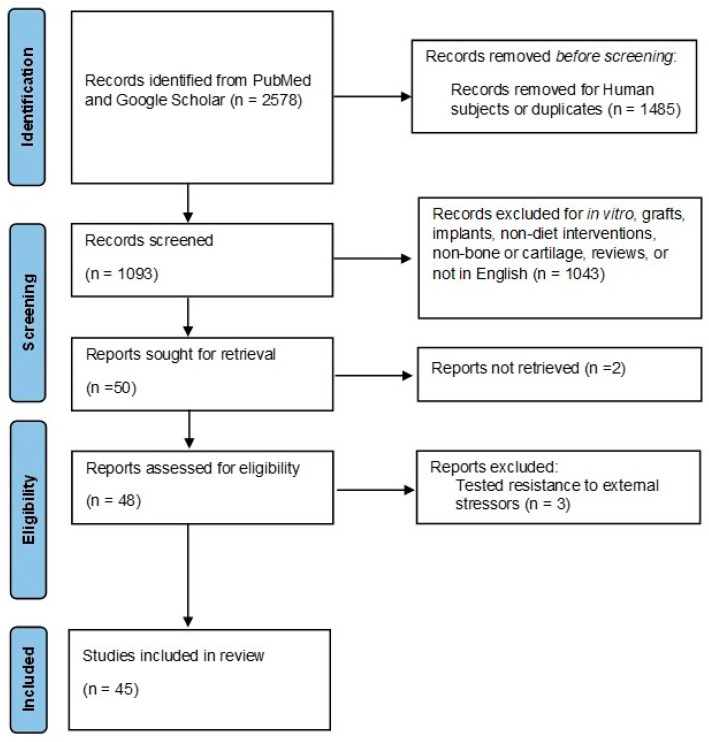
Flow chart of literature search and selection.

Studies were screened in two stages. Initially, the authors reviewed article titles and abstracts for inclusion and exclusion criteria. Articles that met the inclusion criteria were sought for full-text retrieval and assessed for eligibility. Articles that met inclusion criteria but were not available for full-text retrieval or tested external stressors, such as bacterial or viral infections, were further excluded. Figure 2 shows a flow chart of the literature search and selection.

### 2.4. Data Extraction

Silicon type, group name, Si intake from diet, Si supplementation amount, animal, and age at the start were extracted from each study to the extent these data were reported. Silicon type was extracted as reported, and treatments were sorted and standardized into Deficient, Control, or Si-supplemented. Additionally, mineral and bone outcomes were summarized and recorded. Dietary Si intake, Si supplementation amount, and amount of Si standardized on a kg BW basis were calculated based on the information provided. If bodyweight or food intake data were not reported or were unable to be retrieved or estimated by species standards, calculations were not performed, and those treatments were excluded from further analysis. If daily dietary Si intakes were not provided or were unable to be calculated, these treatments were also excluded; however, if dietary Si intake was provided on a mg/kg BW basis, this value was included in the analysis of standardized Si but not dietary Si intake.

### 2.5. Analysis

For clarity, experimental groups or treatments from each included study will be referred to as “treatments”, and the assigned terms—Deficient, Control, or Si-supplemented—will be referred to as “groups”. The means and standard deviations of dietary Si intake and standardized Si for Deficient, Control, and Si-supplemented groups was tabulated overall via JMP 16 (Location), while the mean (±SD) Si supplementation amount was calculated only for the Si-supplemented group. Counts were performed for Si forms and research species. Additionally, Si treatments were noted either as having “No Effect” or a “Positive Effect” on bone or mineral measures, to attempt to determine an effective standardized Si dose. Outliers were explored via interquartile range calculations, and data points were excluded if they were more than three times the interquartile range. A Mann–Whitney U test was conducted to compare dietary Si intake and standardized Si amounts between Control and Si-supplemented groups as well as between No Effect and Positive Effect doses.

## 3. Results

Table 1, Table 2 and Table 3 show the studies grouped by animals included for this literature review along with summary information and results. The counts for Si forms and research species are as follows: Sodium metasilicate was the most reported form (n = 20), followed by sodium zeolite A (n = 11). The remaining forms included monosilicic acid, orthosilicic acid, aluminosilicates, silicon–collagen complex, chelated silica, tetraethyl-orthosilicate, monomethyl-silanetriol, and silanol, but these forms were only used in four or fewer groups or studies. Rats were the most common animal studied (n = 18 treatments), followed by chicks (n = 13 treatments) and horses (n = 6 treatments), with mice (n = 4 treatments), calves (n = 2 treatments), pigs (n = 2 treatments), and turkeys (n = 1 treatment) making up the rest of the experiments. Two papers [26,36] each included two experiments with two different species. Overall mean ± standard deviations, as well as minimums, maximums, and medians for total standardized Si, Si supplementation amounts, and dietary Si intake, are presented in Table 4. Of the 45 articles included in this review, only 20 contained enough information to calculate a standardized Si dose to kg BW, while 22 provided enough information for estimated daily dietary intake and 16 for estimated doses per day. There was not enough information to report these values for treatments labeled as Deficient. There were differences in sums of dietary Si intake and standardized total Si between Control and Si treatments (*p* < 0.01). Additionally, there was also a difference in the sums of standardized Si and daily Si doses between “No Effect” and “Positive Effect” treatments (*p* < 0.05 for both variables; Table 5), with “Positive Effect” doses and standardized Si (116 mg and 139 mg/kg BW, respectively) being substantially higher than “No Effect” (12 mg and 73 mg/kg BW, respectively). 

## 4. Discussion

Value reporting varied dramatically across the included studies, making this review difficult. Nearly half the studies left out animal weights, amount of food or water consumed, or the nutrient profiles of the basal diet. Without this information, the total amount of Si could not be standardized to kg BW, limiting the ability to translate amounts and results to other species, including humans.

The age of the animal at the time of Si supplementation could impact the influence of Si on bone and cartilage outcomes. Early studies examined deficiencies versus Si-adequate diets in chicks during growth and demonstrated the essentiality of Si for bone and cartilage development [1,3,4,6,37]. However, when later experiments did not use purified diets with Si removed and usually studied Si supplementation to already adequate diets, results were mixed even in growing animals.

Sodium metasilicate was the most reported form of Si used for supplementation, likely because many of the early studies with chicks and rats repeatedly used this form to demonstrate the importance of Si for skeletal development. However, these Si species contained metals like Al, which made it difficult to distinguish Si influence on bone versus its ability to suppress Al absorption.

Standardized Si intakes ranged from 0.003 to 863 mg/kg BW. One aim of this review was to determine an “effective dose” on a mg per kg BW basis in order to translate results across species. Unfortunately, due to the lack of critical information reported in many of the studies included in this review, this dose may not be able to be determined on a conclusive basis. Sums of standardized Si intakes in studies demonstrating positive effects on bone and/or mineral metabolism did rank higher in the Mann–Whitney U test than standardized Si intakes in studies showing no effects on bone or mineral metabolism. The difference between these two groups indicates that there may be a level above essentiality at which supplemented Si positively influences these outcomes, though some studies have reported no greater outcomes with increased supplementation [15,26,40,43,45,55]. Previous experiments that have achieved improvements in bone or cartilage quality in adult animals [5,15,27,48,49,53,54,55] fed Si in large amounts, which, when expressed on a per kg BW basis at 18 to 462 mg Si/kg BW, is difficult to translate into use with large animals. Supplemented mature horses receiving a total of 54 mg Si/d or 0.1 mg Si/kg BW had no change in collagen metabolism in synovial fluid, indicating that cartilage turnover remained unaffected, and lameness did not improve [61]. Once expressed on a kg BW basis, this amount is much lower than previously successful experimental levels of Si. Based on current data gathered by this review, it appears that standardized Si intake above 139 mg/kg BW/d may positively influence bone or mineral metabolism outcomes; this is over double the current estimated human intake on a whole-body basis.

Even without an “effective dose”, the standardization of intakes would facilitate research and results translation across species. The current recommendation for human Si intake for bone health is 25 mg/d [23,33]. If a human weighing 65 kg consumed this amount of Si, it would only be around 0.4 mg/kg BW—this is a small amount, even for control treatments in this review. Additionally, the higher end of Si intakes may not be feasible for large animals, including humans. If a human weighing 65 kg consumed the mean standardized Si intake from Si treatments, this would be 7.8 g Si, eclipsing the recommendation from the literature for this mineral as well as the daily recommended allowance for macrominerals like calcium [62]. Supplementing at these amounts would likely alter the metabolism of other crucial minerals [24,27,43,55] and would likely be difficult, if not impossible, to achieve in humans.

Silicon sources for humans include water and diet [22]. Plant-based foods contribute a significant amount of Si, with the largest contribution coming from cereals and cereal products, especially oat bran and oat cakes, which contain roughly 23.4 and 18.3 mg Si/100 g [63]. Silicon in fruits and vegetables ranges from non-detectable to 16.6 mg/100 g. Water, especially mineral water, may contain up to 40 mg Si/L [22]. Even at these amounts, humans will likely struggle to achieve experimental levels of Si, especially if the threshold for consistent positive outcomes is at 139 mg Si/kg BW/d. For a 65 kg human, this amount would be equivalent to eating roughly 38.6 kg of oat bran or drinking 225 L of water. If this threshold is unachievable via normal diet and water intake, supplementation would likely be necessary.

A recent review examining Si intake and bone mineral density in humans came to similar results as this current review [33]. The upper intakes noted in that review were around 40 mg/d for adults, which would be on the low side of Si intakes for studies included in the current review. Supplementation studies in humans included in the recent review included doses from 3 to 86 mg Si per day, in addition to a likely adequate diet. During supplementation, these amounts produced little to no effect on bone or mineral parameters, and these amounts were similar to treatments marked as “No Effect” in the current review, indicating that far greater amounts may be necessary to influence bone health in adults. While the previous review did not note average BW in its included studies, the supplemental and typical Si intakes were likely much lower on a per kg BW basis than experimental amounts in animals, as the animal studies included in the current review averaged 120 mg Si/kg BW.

Limitations of the current review include the lack of effect size, bias estimations for meta-analysis, and the small number of studies from which data could be extracted. While excluding data due to missing information meant several studies were eliminated from final calculations and analysis, the authors felt that estimations of missing data would add to the uncertainty of an already limited data set. Additionally, the authors were unable to explore the effects of Si type and age at the start of supplementation on bone and mineral outcomes due to the previously noted difficulties of inconsistent data reporting.

It is likely that many of the experimental amounts of Si providing some benefits in animals will prove difficult to translate to humans. Providing Si on top of an already adequate diet likely does not influence bone health measures in healthy adult animals, despite changes to mineral metabolism, but a lack of Si during growth is associated with negative changes to bones and cartilage. The lack of data provided by the literature made definitively determining an effective threshold of supplementation for skeletal health difficult, though it appears that Si consistently positively influences bone and mineral metabolism measures at around 139 mg/kg BW/d. Future studies should examine this threshold more directly to determine if positive bone or mineral effects can be consistently achieved above it. Ultimately, studies should standardize supplemental or dietary intakes of this micromineral to kg BW for better study replication and translation.

## Figures and Tables

**Table 1:** Brief summaries of studies using chicks or turkeys included in this review. ^1^ Article contained multiple experiments, and amounts for each group were averaged based on the number of experiments in which they appeared. X indicates information not applicable; “-” denotes missing or not enough information to calculate.

Reference	Silicon Form	Group	Daily Intake from Diet	Silicon Dose per Day	Total Silicon mg/kg BW	Animal	Age at Start	Results
Carlisle, 1972 [1]	Sodium metasilicate	Control	1 ppm	X	-	Chicks	1 day	Reduced growth rate, shorter leg bones with smaller circumferences and thinner cortices in control, control tibias and femurs fracture more easily
		Supplemented	100 ppm					
Carlisle 1976 [6]	Sodium metasilicate	Control	<3 ppm	0 mg	-	Chicks	1 day	Better growth, higher hexosamine content and percent in articular cartilage, greater silicon content in comb, less water content in tibia and femur with supplementation, no difference in percent ash
		Supplemented		100 ppm				
Carlisle, 1980a [4]	Sodium metasilicate	Control	1 ppm	X	-	Chicks	1 day	Greater percentage and total amount of hexosamine and greater percentage of collagen in tibias from supplemented vs control, Si-deficient tibias had lesions and changes in epiphyseal cartilage especially in proliferative zone
		Supplemented	250 ppm					
Carlisle, 1980b [3]	Sodium metasilicate	Control	1 ppm	X	-	Chicks	1 day	Si-deficient skulls had less trabeculae and calcification, reduced collagen content
		Supplemented	250 ppm					
Carlisle, 1981 [37]	Sodium metasilicate	Control	1 ppm	X	-	Chicks	1 day	Skull abnormalities in Si-deficient chicks from less collagen concentration in bones
		Supplemented	250 ppm					
Merkley and Miller, 1983 [38]	Sodium metasilicate	Control	-	X	-	Chicks	1 day	Humeri strength decreased during immobilization in control but remained similar to unrestricted humeri strength with metasilicate
		Sodium metasilicate		74 ppm				
Watkins, Vagnoni, and Southern, 1989 [39]	Sodium zeolite A	0%	-	0 mg	-	Chicks	4 days	SZA with excess Ca decreased weight gain and tibia ash
		0.75%		90.3 mg				
Elliot and Edwards, 1991 [40]	Sodium metasilicate	Basal ^1^	0.01 mg	0 mg	0.02	Chicks	1 day	High silicon inclusion reduced feed efficiency, no difference in tibial ash
		25 ^1^		0.20 mg	0.66			
		50 ^1^		0.46 mg	1.53			
		150 ^1^		1.44 mg	4.78			
		250 ^1^		2.72 mg	7.62			
Watkins and Southern, 1992 [41]	Sodium zeolite A	0% SZA	-	X	-	Chicks	4 days	Plasma Ca or alkaline phosphatase unaffected by SZA, reduction in plasma P but increase in tibia Mn, Zn, Cu, and Al with SZA
		0.75% SZA						
Scheidler, 1993 [42]	Aluminosilicates	Control	-	X	-	Chicks	1 day	Novasil increased bone ash, Ethacal decreased bone ash, supplementation decreased serum Cl
		Ethacal	163 mg		279			
		Novasil	288 mg		506			
		Perlite	357 mg		543			
		Zeobrite	333 mg		514			
Kayongo-Male and Julson, 2008 [26]	Tetraethyl-orthosilicate	Groups based on supplemented Si levels		0 ppm	-	Turkeys	1 day	Moment of inertia and plasma calcium lower with high supplementation, no differences in other physical or mechanical properties
				135 ppm				
				270 ppm				
				540 ppm				
Sgavioli et al., 2016 [30]	Not given	0 mg Supplement	-	X	-	Chicks	1 day	Si supplementation had no effect on bone density or breaking strength, bone ash, phosphorus, zinc, and manganese increased without increasing bone calcium
		0.5 mg Supplement		244 mg	150			
		1.0 mg Supplement		488 mg	300			
		1.5 mg Supplement		740 mg	450			
Scholey et al., 2018 [32]	Monomeric silicic acid	Control ^1^	55.8 mg	X	114	Chicks	1 day	Improved tibia breaking strength and tibial Si at 1000 mg/L supplementation, foot and tibia ash increased in the 500 mg/L, no other significant differences in bone measures
		200 mg/L		16.2 mg	138			
		500 mg/L		39.5 mg	166			
		1000 mg/L ^1^		79.5 mg	280			
Pritchard et al., 2020 [43]	Orthosilicic acid	Control	2.9 mg	X	4.1	Chicks	1 day	Supplementation reduced serum boron and increased serum calcium; bone density, morphology, and strength measures were similar among groups
		Normal		133 mg	147			
		High		804 mg	863			

**Table 2:** Brief summaries of studies using rats or mice included in this review. X indicates information not applicable; “-” denotes missing or not enough information to calculate.

Reference	Silicon Form	Group	Daily Intake from Diet	Silicon Dose per Day	Total Silicon mg/kg BW	Animal	Age at Start	Results
Schwarz and Milne, 1972 [2]	Sodium metasilicate	Control	<5 ppm	X	-	Rats	20 days	Improved growth rates across two different diet compositions, improved incisor pigmentation and skull bone structure
		Supplemented		500 ppm				
Najda et al., 1993 [24]	Sodium metasilicate	Control	-	0 mg	-	Rats	2 months	Supplementation increased serum Ca and tissue Mg
		Supplemented		0.7 mg/g BW				
Hott et al., 1993 [44]	Silanol	Sham operated	-	X	-	Rats	3 months	Silanol decreased osteoclast surface and number of osteoclast, higher dose increased mineral apposition rate and bone formation rate, no effect on the periosteal apposition rate with silanol
		Ovariectomized		X	-			
		Ovariectomized + low silanol		0.1 mg/kg	-			
		Ovariectomized + high silanol		1.0 mg/kg	-			
Firling et al., 1996 [45]	Sodium zeolite A	Normal Ca, 30 mg SZA/kg BW	-	X	9.9	Rats	-	No effect of SZA on cortical or cancellous bone formation and mass
		Normal Ca, 100 mg SZA/kg BW	-		33			
		Normal Ca, 500 mg SZA/kg BW	-		165			
		Low Ca, 0 mg SZA/kg BW	-		0			
		Low Ca, 125 mg SZA/kg BW	-		41.3			
		Low Ca, 617 mg SZA/kg BW	-		204			
Rico et al., 2000 [46]	Sodium metasilicate	OVX	-	X	-	Rats	100 days	Attenuated bone loss in vertebra and femur in OVX + Si
		OVX-Sham	-					
		OVX + Si	50 g/100 g diet					
Seaborn and Nielsen, 2002 [47]	Sodium metasilicate	−Si	2.3 μg/g diet	0 μg/g	-	Rats	21 days	Tibial hydroxyproline lower and decreased liver ornithine aminotransferase in deficient rats
		+Si		10 μg/g				
Seaborn and Nielsen, 2002 [29]	Sodium metasilicate	−Si	2.3 μg/g	0 μg/g	-	Rats	21 days	Depressed growth, lower plasma Si, and lower femoral Ca concentrations in −Si, Lower alkaline phosphatase in +Si
		+Si		25 μg/g				
Calomme et al., 2006 [48]	Orthosilicic acid	Sham	-	X	-	Rats	9 months	OSA supplementation partially reversed the decrease in Ca excretion seen in OVX, tended to reduce bone turnover, increased total femoral BMC and BMD, marginally increased total lumbar BMD
		OVX		X	-			
		OVX-Si		1 mg/kg BW	-			
Bae et al., 2008 [49]	Sodium metasilicate	Sham	0.09 mg	X	0.3	Rats	17 weeks	Supplementation increased femur and tibia BMD and serum CTx and decreased urinary Ca and P excretion compared to OVX
		OVX	0.11 mg	X	0.4			
		OVX-Si	0.10 mg	6.21 mg	65.4			
Jugdaohsingh et al., 2008 [50]	Sodium silicate	Si-Deprived	0.05 mg	X	0.2	Rats	3 weeks	Serum Si concentrations and urinary excretion lower in Si-deprived vs Si-supplemented, tibia Si lower in Si-deprived and Si-supplemented than Normal, Si-deprived showed reduced bone growth plate thickness, increased in chondrocyte density and lower tibia phosphorus concentrations
		Si-Supplemented	0.05 mg	53.2 μg/g water	4.1			
		Normal	5.46 mg	X	18.5			
Maehira et al., 2008 [51]	Sodium metasilicate/ Monosilicic acid	Tap Water (Control)	9.4 μg	X	-	Mice	-	DW and Si improved bone bio- chemical indices such as femoral weight, mineral and collagen content, and marker enzymes of bone formation and resorption as well as mechanical properties as compared to TW
		Deep Sea Water	15.7 μg					
		Surface Sea Water	9.9 μg					
		Tap + 200 ppm Si	20.0 μg					
Kayongo-Male and Julson, 2008 [26]	Tetraethyl-orthosilicate	Groups based on supplemented Si levels	5 ppm	0 ppm	-	Rats	-	Moment of inertia lower and trend for reduced plasma Mg with supplementation, no other physical or mechanical differences
				500 ppm				
Kim et al., 2009 [15]	Sodium metasilicate	Low Ca	0.08 mg	X	0.39	Rats	6 weeks	Supplementation increased BMD in femur and tibia of Ca-deficient ovariectomized rats, lower serum CTX in Si low calcium group but higher CTX in adequate calcium group
		Low Ca + Si Supplement	80.1 mg		398			
		Adequate Ca	0.09 mg		0.42			
		Adequate Ca + Si Supplement	81.9 mg		408			
		High Ca	0.08 mg		0.41			
		High Ca + Si Supplement	90.9 mg		443			
Maehira et al., 2009 [17]	Sodium metasilicate/ Monosilicic acid	Control (CT)	0.84 μg	X	-	Mice	1 month	Femoral collagen content increased while OHProline urinary excretion decreased in Si, increased strength and structural stiffness in Si
		CT + Si	213.1 μg					
		Coral Sand (CS)	2.12 μg					
		Fossil Stony Coral (FCS)	1.26 μg					
		Fish Bone (FC)	2.17 μg					
		Eggshell (EC)	0.94 μg					
Kim et al., 2014 [27]	Sodium metasilicate	Control	22.97 μg	X	0.55	Mice	9 weeks	No difference in BMD in femur and tibia, adjusted BMD for final BW higher in Si50, femur area was higher in Si50 and Si150 than in control, supplementation decreased Mg retention without changing Ca retention, and decreased ALP
		Si50		1958 μg	48.5			
		Si100		2877 μg	74.6			
		Si150		3636 μg	89.4			
Jugdaohsingh et al., 2015a [5]	Monomethyl-silanetriol	Group 1	16.5 mg	X	44.6	Rats	2 months	Si supplementation increased fasting serum and tissue Si concentrations, trend for serum OC concentration in female rats to show a dose-response increase, strong significant associations between serum Si concentrations and bone quality in female rats
		Group 2		2.98 mg	53.4			
		Group 3		16.1 mg	90.7			
Jugdaohsingh et al., 2015b [7]	-	Groups divided by age	628 μg/g diet + 3.9 μg/mL water	X	-	Rats	23 days	Higher Si concentrations (depending on age) found in connective tissues with highest amount found in the 3 or 5 wk old rats, Si decreased with age except in skin, decreases occurred pre-puberty and stabilize in adulthood, higher serum Si in younger animals, Total Si increases with growth of organ, linear association with bone, difference in total body Si between weanling and adult is less than 100 μg
Bu, Kim, and Choi, 2016 [52]	Metasilicate	Control	0.09 mg	X	0.36	Rats	7 weeks	Si supplementation unable to restore ovariectomy induced BMD decreases with Ca-replete diet, OVXVHSi increased OPG expression and decreased RANKL/OPG ratio in mRNA expression comparable to levels of sham-controls
		OVXNSi (OVX control)	0.09 mg		0.36			
		OVXHSi	4.29 mg		17.8			
		OVXVHSi	12.8 mg		53.1			
Qi and Zheng, 2017 [53]	Sodium metasilicate	OVX	-	X	-	Rats	3 months	Si improved BMD, bone histological and serum biochemical parameters in ovariectomized rats
		OVX-Si		5.44 mg	20			
		OVX-GEN-Si		5.15 mg	20			
Chen, Zheng, and Qi, 2019 [54]	Sodium metasilicate	Control	0.06 mg	X	-	Rats	3 months	Si improved BMD, bone histological and serum biochemical parameters in ovariectomized rats
		Supplemented		4.65 mg	20			
Kim and Choi, 2021 [55]	Sodium metasilicate	Low Ca + Adequate Si	0.07 mg	X	0.4	Rats	6 wks	Si supplementation decreased serum CTx and increased serum Mg in low Ca, reduced BMD at femur and tibia in high Ca, and increased tibia strength in adequate Ca
		Low Ca + High Si	7.28 mg		38.4			
		Adequate Ca + Adequate Si	0.08 mg		0.4			
		Adequate Ca + High Si	7.44 mg		38.7			
		High Ca + Adequate Si	0.07 mg		0.4			
		High Ca + High Si	7.62 mg		40.2			
Bychkov et al., 2022 [36]	Chelated silica	Control	-	X	-	Mice and Rats	12 wks and 4 wks	Increase in Alkaline phosphatase in chelated silica supplemented mice; otherwise, no differences between silicon-chelated supplemented and control animals
		Chelated Silica		6 mg (Mice)				
				24 mg (Rats)				

**Table 3:** Brief summaries of studies using horses, pigs, or calves included in this review. X indicates information not applicable; “-” denotes missing or not enough information to calculate.

Reference	Silicon Form	Group	Daily Intake from Diet	Silicon Dose per Day	Total Silicon mg/kg BW	Animal	Age at Start	Results
Ward et al., 1991 [56]	Sodium zeolite A	0% SZA	-	X	-	Pigs	31 days	SZA increased serum alkaline phosphatase and liver and bone Zn content, decreased serum Ca and inorganic P concentrations
		0.5% SZA	3080 mg		122			
Frey et al., 1992 [57]	Sodium zeolite A	0% SZA	-	0 mg	-	Horses	6 months	Increased plasma silicon concentrations with supplementation, gain in BMC for first 56 days greatest in 2.0% SZA but no differences among treatments in BMC over the course of the study
		0.66% SZA		4.3 mg				
		1.32% SZA		8.7 mg				
		2.0% SZA		12.5 mg				
Nielsen et al., 1993 [11]	Sodium zeolite A	0% SZA	-	0 mg	-	Horses	18 months	Increased plasma silicon concentrations and faster average race times, 1.86% and 2.8% increased distance and training/racing cycles prior to injury
		0.92% SZA		10.3 mg				
		1.86% SZA		20.8 mg				
		2.8% SZA		31.4 mg				
Calome and Vanden Berghe, 1997 [13]	Orthosilicic acid	Control	360 mg	0 mg	4.3	Calves	1 week	Increased Si serum and collagen dermis concentration
		Supplemented	378 mg	17.5 to 70 mg	4.9			
Lang et al., 2001 [58]	Sodium zeolite A	Control	10.8 g	X	-	Horses		Supplemented mares had higher plasma and milk Si concentrations, foals of Supplemented mares had higher plasma Si concentrations but did not influence bone metabolism in foals
		Supplemented	44.3 g					
Lang et al., 2001 [12]	Sodium zeolite A	Control	9.25 g	X	27.0	Horses	1 year	Higher plasma Si concentrations and lower ICTP in Si treated group, no differences for OC or PYD
		Si Treated	30.57 g		87.2			
O’Connor et al., 2007 [25]	Sodium aluminum silicate/orthosilicic acid	Control	874 mg	X	1.7	Horses	10 years	SA increased Si excretion and calcium retention and apparent digestion, OSA increased Ca and B retention, apparent B and Si digestion, plasma Si, and tended to increase Si retention
		SA		124 mg	1.9			
		OSA		137 mg	2.2			
Frantz et al., 2008	-	Control	0 mg	X	0	Pigs	-	Si Diet had lower overall osteochondrosis incidence scores than Control
		Si Diet	2790 mg		46.1			
Turner et al., 2008 [59]	Sodium zeolite A	Control (CO)	2.7 g	X	41.2	Calves	3 days	No differences in OC concentrations, OC:DPD ratio, bone architecture, mechanical properties, or glycosaminoglycan concentration in cartilage or synovial fluid CO had lower DPD concentrations, SS had greater cortical bone and articular cartilage aluminum content
		Supplemented (SS)		6.5 g	138			
Pritchard et al., 2020 [60]	Silicon-collagen	Control	1.8 mg	X	0.003	Horses	13 years	No differences
		Supplemented		52.7 mg	0.1			

**Table 4:** Minimums (Min), maximums (Max), medians, and means (±SD) of daily dietary Si intake, Si supplementation dose, and standardized total Si intake from control (Con) and supplemented (Si) groups from animal studies included in this review.

	Min		Max		Median		Mean ± SD	
	Con	Si	Con	Si	Con	Si	Con	Si
Daily Dietary Si Intake (mg)	0.0008	0.01	17	2790	0.08	7.4	1 ± 4	175 ± 534
Daily Si Supplementation Dose (mg)	-	0.2	-	804	-	16.1	-	81 ± 187
Standardized Total Si Intake (mg/kg BW)	0.003	0.1	114	863	0.41	47.3	12 ± 27	120 ± 189

**Table 5:** Mean (±SD) amounts of daily Si supplementation doses and standardized total Si intake from studies resulting in “No Effect” or “Positive Effect” on bone or mineral metabolism, excluding controls.

	No Effect	Positive Effect
Daily Si Supplementation Dose (mg)	12 ± 21	116 ± 223
Standardized Total Si Intake (mg/kg BW)	73 ± 140	139 ± 214
